# Suppression of miR-195 attenuates oxygen–glucose deprivation/reperfusion-induced BBB destruction, possibly via targeting BCL2L2

**DOI:** 10.3389/fnins.2026.1754128

**Published:** 2026-02-10

**Authors:** Mingyan Dong, Lihui Huang, Qiaohong Zhang, Shuchen Zhu, Yicui Piao, Zijie Liu

**Affiliations:** National Cancer Center/National Clinical Research Center for Cancer/Cancer Hospital and Shenzhen Hospital, Chinese Academy of Medical Sciences and Peking Union Medical College, Shenzhen, China

**Keywords:** apoptosis, BBB, BCL2L2, miR-195, OGD/R

## Abstract

**Background:**

MicroRNAs (miRNAs) are highly expressed in the brain and represent promising therapeutic targets for the treatment of ischemic stroke. Previous studies have shown that microRNA-195 (miR-195) is associated with apoptosis and is significantly upregulated in the serum of patients with ischemic stroke. We aimed to confirm the role of miR-195 in brain microvascular endothelial cell (BMEC) apoptosis and blood–brain barrier (BBB) integrity.

**Materials and methods:**

bEnd.3 cells were exposed to oxygen–glucose deprivation/reperfusion (OGD/R). RT-qPCR was used to determine the relative expression of miRNA-195. Bioinformatics analysis using the TargetScan database predicted BCL2L2 as a potential target of miR-195. A BBB model was constructed by culturing bEnd.3 cells in the upper Transwell chambers. Transepithelial/transendothelial electrical resistance (TEER) and the fluorescein isothiocyanate (FITC)-dextran assay were used to assess BBB permeability. Immunofluorescence staining for caspase-3, TdT-mediated dUTP nick end labeling (TUNEL) staining, and flow cytometric analysis were used to measure bEnd.3 cell apoptosis. Tight junction proteins (TJPs) expression was determined using western blot analysis.

**Results:**

miR-195 expression was upregulated in the *in vitro* OGD/R model. miR-195 mimics exacerbated cellular apoptosis and BBB disruption following OGD/R, whereas the miR-195 inhibitor alleviated OGD/R-induced apoptosis and BBB impairment. Overexpression of miR-195 significantly reduced BCL2L2 expression, and luciferase reporter assays confirmed that miR-195 directly binds to BCL2L2. Co-transfection of miR-195 mimics and BCL2L2 partially reversed the effects of miR-195 mimics on cell survival and barrier function.

**Conclusion:**

Our results suggest that the miR-195/BCL2L2 axis plays a critical role in the regulation of bEnd.3 cell apoptosis. Modulation of miR-195 may represent a novel therapeutic strategy for targeting BMEC apoptosis in ischemic stroke.

## Introduction

Recently, ischemic stroke research has shifted its focus from neurons to the neurovascular unit (Wang et al., 2021). The blood–brain barrier (BBB), a highly selective barrier within the neurovascular unit, comprises brain microvascular endothelial cells (BMECs) in close communication with pericytes and astrocytes (Schaeffer and Iadecola, 2021). The BBB is an important target in cerebral ischemia/reperfusion (I/R) injury (Zhang et al., 2024). BMEC apoptosis and disruption of tight junction proteins (TJPs) play important roles in I/R injury after ischemic stroke (Yang et al., 2019). Attenuating BMEC apoptosis and preserving TJPs are beneficial for stabilizing the BBB after cerebral ischemia.

MicroRNAs (miRNAs) are noncoding RNAs approximately 18–22 nucleotides in length that play crucial roles in various fundamental biological processes, including cell proliferation, differentiation, and apoptosis (Pozniak et al., 2022; Jorge et al., 2021; Galagali and Kim, 2020). Many miRNAs exhibit altered expression levels after ischemic stroke and have been proposed as diagnostic markers (Payne et al., 2023; Bsat et al., 2021; Eyileten et al., 2025). Several miRNAs are involved in regulating the permeability of the BBB after cerebral I/R injury (Payne et al., 2023; Eyileten et al., 2025; Gugliandolo et al., 2021). The level of microRNA-195 (miR-195) in serum collected during the acute phase of acute stroke patients was significantly elevated (Zhai et al., 2020). miR-195 has been shown to regulate cell apoptosis by binding to target genes (Lin et al., 2021; Liu et al., 2021; Gao et al., 2022).

BCL2L2 is a crucial apoptosis regulatory protein that functions as a downstream target of miRNAs to modulate apoptotic processes (Yu et al., 2021; Zhang et al., 2016). After searching the online miRNA TargetScan database^1^, we found that BCL2L2 is a potential target of miR-195. Reducing the expression of miR-195 may exert a protective effect against I/R injury. Therefore, this study was designed to investigate the regulatory role of the miR-195/BCL2L2 molecular axis in I/R-induced BBB injury.

## Materials and methods

### Cell culture, oxygen–glucose deprivation/reperfusion (OGD/R) treatment, and plasmid transfection

Cerebral microvascular endothelial cells (bEnd.3 cells; Bioleaf Biotech, Shanghai, China) were cultured in an incubator (5% CO2, 95% O2), with saturated humidity at 37 °C. After reaching 80% confluence, bEnd.3 cells were prepared for transfection. miR-195 mimics, miR-195 inhibitors, and a BCL2L2 overexpression plasmid (oe-BCL2L2) were purchased from RiboBio (Guangzhou, China). We used Lip2000 (Invitrogen, USA) according to the manufacturer’s instructions for plasmid transfection. In addition, 48 h after transfection, cells transfected with the overexpression plasmid were harvested. The cells were placed in a tri-gas incubator containing 1% O2, 5% CO2, and 94% N2 at 37 °C in glucose-free Dulbecco’s Modified Eagle Medium for 6 h, followed by reperfusion in normal medium with glucose under95% air and 5% CO2for 6 h. Cells were collected for subsequent biochemical analysis.

### RT-qPCR

Total RNA was extracted from cells and tissues using TRIzol reagent (Thermo, USA). The RNA was then reverse-transcribed into cDNA using the PrimeScript RT Reagent Kit (TaKaRa Bio, Japan). Afterward, RT-PCR was performed using the SYBGREEN PCR Master Mix Kit (ComWin, Beijing, China). The Ct value for each well was recorded. U6 was used as the internal reference for miR-195. The fold changes were calculated using relative quantification (the 2-△△Ct method). The primer sequences used were as follows:

miR-195F: 5′-TAGCAGCACAGAAATATTGGC-3′
R: 5′-GCTGTCAACGATACGCTACGTA-3′
U6F: 5′-CTCGCTTCGGCAGCACA-3′
R: 5′-AACGCTTCACGAATTTGCGT-3′

### Western blotting

Total proteins were extracted from cultured cells using RIPA lysis buffer (Abiowell, China). Protein concentrations were measured using a BCA protein assay kit (Abiowell, China). Extracted proteins were separated using 10% SDS-PAGE and then electrotransferred to PVDF membranes. The membranes were blocked with TBST containing 5% non-fat milk for 90 min and then incubated overnight with primary antibodies against occludin (1:5000, Proteintech), claudin-5 (1:5000, Proteintech), BCL2L2 (1:1000, Proteintech), and *β*-actin (1:5000, Proteintech). Afterward, the membranes were washed and incubated for 1 h with secondary antibodies at room temperature. Immunocomplexes on the membrane were visualized using enhanced chemiluminescence reagent (K-12045-D50, Advansta, USA). The ImageJ software (LOCI, Madison, WI, USA) was used for the quantitative analysis of the western blot results.

### Flow cytometric analysis of apoptosis

bEnd.3 cells were treated with overexpression plasmids for 48 h and subjected to oxygen–glucose deprivation/reperfusion (OGD/R). Cell apoptosis was analyzed by flow cytometry using the Annexin V-APC apoptosis detection kit (KeyGEN, Nanjing, China) according to the manufacturer’s instructions. Briefly, cells were collected after digestion with ethylenediaminetetraacetic acid (EDTA)-free trypsin, washed, and resuspended in PBS. The cell suspension was mixed with 5 μL Annexin V-APC and 5 μL propidium iodide, then incubated at room temperature for 10 min in the dark. The cells were subsequently analyzed by flow cytometry, and the data were processed using the FlowJo software.

### Construction of an *in vitro* BBB model

bEnd.3 cells were seeded on type I collagen-coated 24-well Transwell culture inserts (0.33 cm2, 0.4-μm pore size; Corning, BD Biosciences). A BBB model was established once the cells formed a confluent monolayer. The barrier function of the BBB model was evaluated by determining transepithelial/transendothelial electrical resistance (TEER) and using the fluorescein isothiocyanate (FITC)-dextran permeability assay.

### Transepithelial/Transendothelial electrical resistance

The barrier integrity of bEnd.3 cells was assessed by measuring TEER using an ERS-2 Voltohmmeter (Millipore, USA). All TEER values were normalized to the area of the membrane (0.33 cm^2^) and corrected for the resistance without cells.

### FITC-dextran permeability assay

BBB model permeability was assessed using the FITC-dextran permeability assay. bEnd.3 cells were seeded onto 24-well Transwell upper chambers (0.33 cm2, 0.4-μm pore size; Corning). After OGD12h/R6h, 1 mg/mL FITC-dextran was added to the upper layer of the chamber. After 1 h, lower chamber samples were collected. The absorbance of the lower chamber samples was measured using a fluorescence spectrophotometer (490 nm excitation and 525 nm emission).

### TUNEL staining

To assess cell apoptosis after OGD/R insult, TdT-mediated dUTP nick end labeling (TUNEL) staining (Yisheng Biotechnology, Shanghai, China) was performed to detect the apoptotic rate. In brief, BMECs were incubated with proteinase K. The terminal deoxynucleotidyl transferase (TdT) and fluorescein-conjugated dUTP were added to the cell samples. Nuclei were counterstained with 4’,6-diamidino-2-phenylindole (DAPI; Wellbio Biotechnology, Shanghai, China). TUNEL-positive cells were analyzed using fluorescence microscopy (Motic BA410E, Xiamen, China).

### Luciferase assay

The luciferase reporter vector pmiR-BCL2L2-WT (wild type) was constructed by cloning BCL2L2 cDNA, which contains the binding site of miR-195. The luciferase reporter vector pmiR-BCL2L2-Mut(mutant) was constructed by inserting the mutant BCL2L2 sequence. According to the manufacturer’s instructions, the luciferase reporter vector was co-transfected with miR-195 mimics into BMECs using Lipofectamine 2000 (Invitrogen). Renilla luciferase reporters were used as an internal control. Luciferase assay was performed after 48 h using the Dual-Luciferase Reporter Assay System (Promega Biotech).

### Caspase-3 activity

Cells cultured in dishes were fixed with 4% paraformaldehyde for 30 min. After washing three times with PBS, the cells were permeabilized with 0.3% Triton X-100 at 37 °C for 30 min, washed again with PBS, and blocked with 5% BSA for 1 h. Subsequently, the cells were incubated with a primary antibody against caspase-3 at 4 °C overnight, followed by incubation with a fluorescently labeled anti-rabbit IgG secondary antibody at 37 °C for 90 min. After three washes with PBS, nuclei were stained with DAPI working solution at 37 °C for 10 min. Images were captured using a fluorescence microscope.

### Statistical analysis

All quantitative data are presented as mean ± standard error of the mean (SEM). Differences were evaluated using one-way ANOVA (with Tukey’s multiple comparisons test) or Student’s *t*-test (two groups). All statistical analyses were performed using GraphPad Prism 10 (GraphPad Software). Statistical significance was set at a *p*-value of <0.05.

## Results

### miR-195 in bEnd.3 cells increased after OGD/R

**Figure 1:**
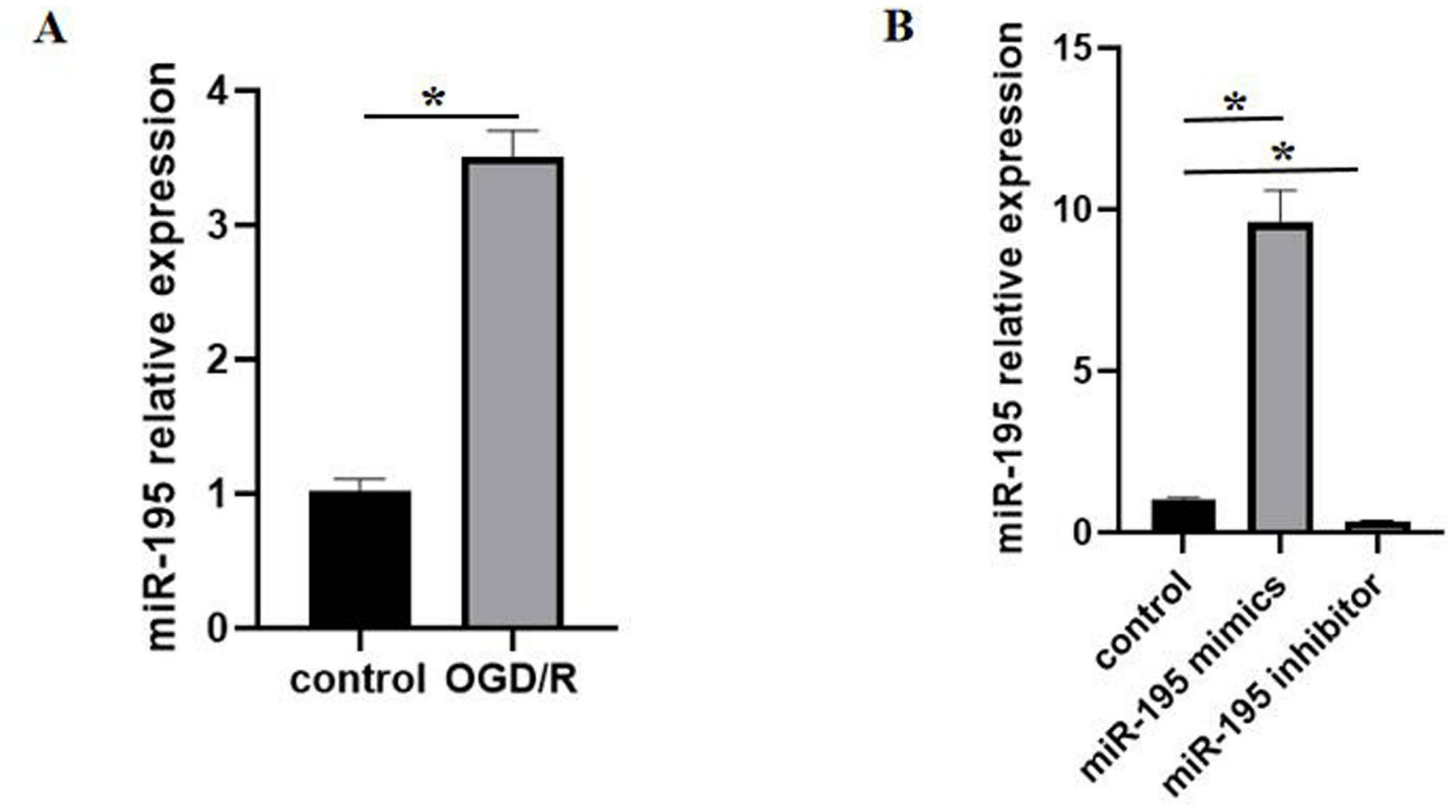
Mi-195 expression was markedly upregulated following OGD/R. **(A)** Relative expression of miR-195 in bEnd.3 cells after OGD/R treatment (*n* = 5, mean ± SEM). **(B)** Relative expression of miR-195 in bEnd.3 cells after transfection with miR-195 mimics or miR-195 inhibitors, as detected by RT-qPCR (*n* = 5, mean ± SEM). *p < 0.05 between the two indicated groups. *n*, number of cell samples.

Previous studies have indicated that altered expression of miR-195 in patients with ischemic stroke may suggest its potential as a diagnostic biomarker (Zhai et al., 2020). Since miR-195 is expressed in BMECs, we employed an OGD/R model to investigate its role in modulating BBB stability during ischemic stroke. RT-qPCR analysis showed that miR-195 in bEnd.3 cells under OGD/R conditions increased significantly compared to the control group (Figure 1A). This finding suggests that the upregulation of miR-195 may be involved in cellular responses to I/R injury. Subsequent experiments demonstrated that transfection with miR-195 mimics markedly enhanced cellular miR-195 expression, whereas transfection with a miR-195 inhibitor significantly reduced its expression level relative to the control group (Figure 1B).

### Downregulation of miR-195 alleviated BBB damage after OGD/R

**Figure 2:**
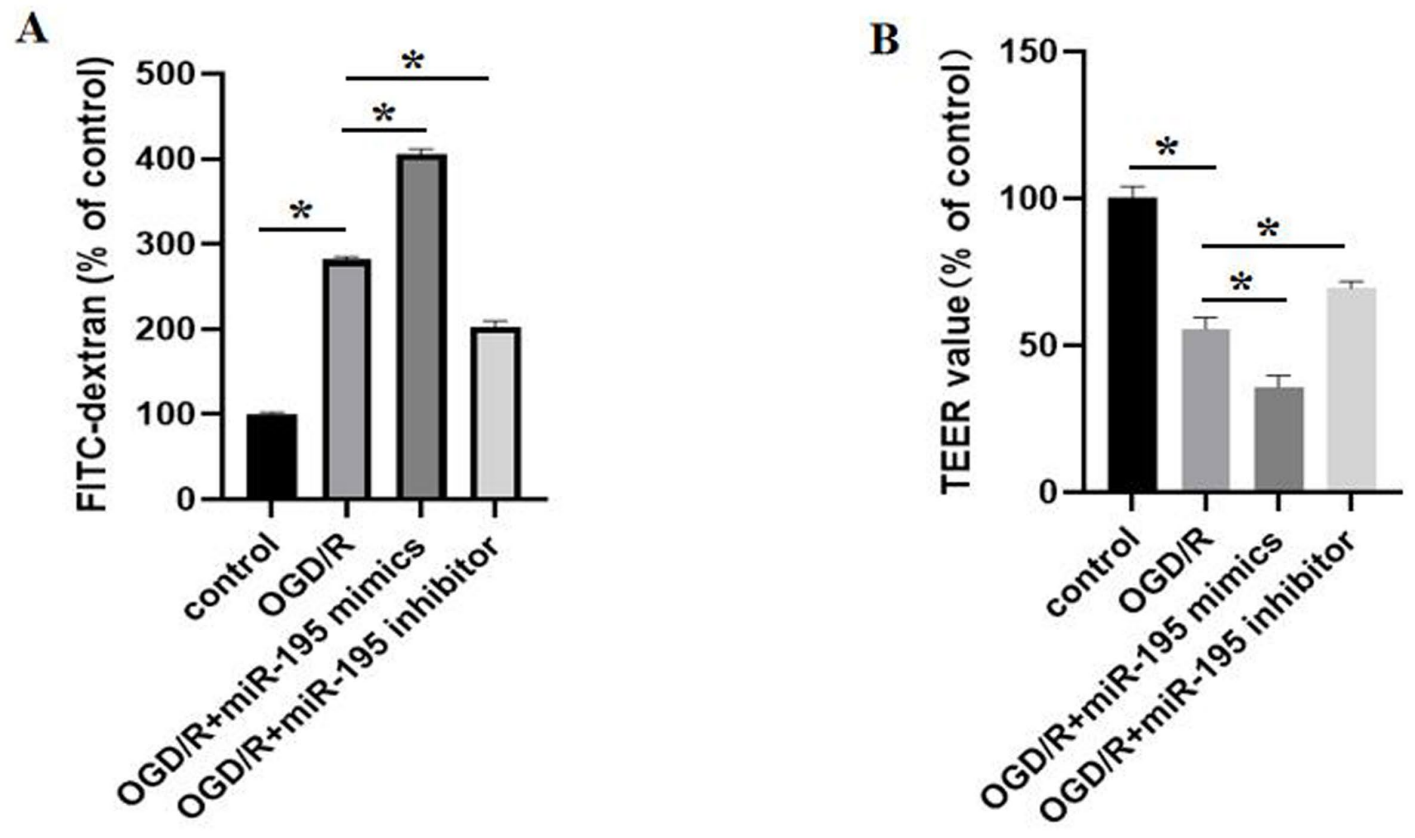
OGD/R affected BBB permeability. MiR-195 inhibitors abolished the effect of OGD/R on BBB integrity, while miR-195 mimics exacerbated the effect of OGD/R on BBB integrity. **(A)** Permeability of bEnd.3 cells was analyzed using fluorescein isothiocyanate (FITC)-dextran (*n* = 5, mean ± SEM). **(B)** TEER of bEnd.3 cell monolayers in the different groups (*n* = 5, mean ± SEM). *p < 0.05 between the two indicated groups. *n*, number of cell samples.

We used the monolayer of the bEnd.3 cells grown on the Transwell insert as an *in vitro* BBB model. To examine the effect of miR-195 expression on cerebral ischemic injury, we transfected the bEnd.3 cells with miR-195 mimics or miR-195 inhibitors, and the effects of miR-195 on BBB function after OGD/R injury was assessed. Permeability of the *in vitro* BBB model was assessed after OGD/R. Relative bEnd.3 cell permeability was evaluated by measuring the permeability of FITC-dextran (40 kDa) across the cell monolayer and by determining TEER values. The FITC-dextran (40 kDa) leakage test showed that the permeability of bEnd.3 cells exposed to OGD/R increased significantly compared to the control group. Compared to the OGD/R group, miR-195 mimics increased cellular permeability, whereas the miR-195 inhibitor reversed this effect (Figure 2A). Consistent with the permeability results, the TEER of the endothelial monolayer exposed to OGD/R was significantly reduced compared to the control group. The miR-195 inhibitor protected the endothelial monolayer from OGD/R-induced TEER reduction, while miR-195 mimics exacerbated the decline in TEER following OGD/R (Figure 2B).

### Downregulation of miR-195 attenuated OGD/R-induced apoptosis in bEnd.3 cells

**Figure 3:**
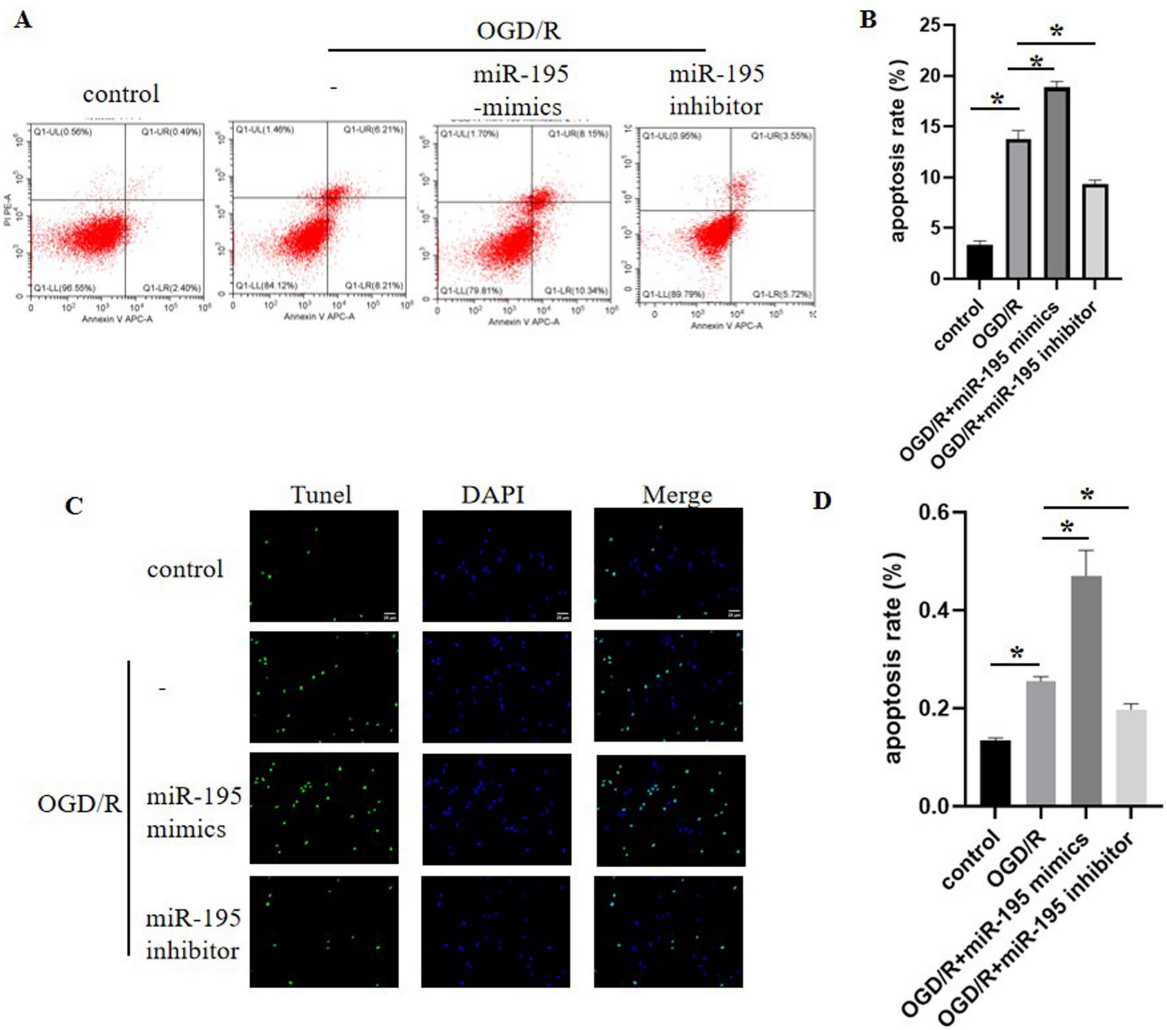
OGD/R induced bEnd.3 cell apoptosis, and this effect was reversed by the miR-195 inhibitor, while miR-195 mimics exacerbated this apoptotic response. **(A)** Annexin V and propidium iodide (PI) staining of bEnd.3 cells, analyzed by flow cytometry to identify apoptotic cells (Annexin V^+^/PI^−^ and Annexin V^+^/PI^+^). **(B)** Quantitative analysis of the apoptotic rate in bEnd.3 cells (*n* = 5, mean ± SEM). *p < 0.05 between the two indicated groups. *n* = number of cell samples. **(C,D)** DAPI/TUNEL double staining of bEnd.3 cells (DAPI: blue, TUNEL: green), and quantitative analysis of TUNEL staining (*n* = 5, mean ± SEM). *p < 0.05 between the two indicated groups. *n*, number of cell samples.

It is well established that apoptosis of BMECs increases after ischemic stroke, leading to enhanced BBB permeability. Annexin V/PI staining analysis showed that the apoptotic rate of bEnd.3 cells was significantly increased after OGD/R compared to the control group. Transfection with the miR-195 inhibitor attenuated OGD/R-induced apoptosis, whereas miR-195 mimics promoted apoptotic cell death (Figures 3A,B). These findings from Annexin V/PI analysis were further confirmed by TUNEL assay (Figures 3C,D).

### BCL2L2 is a miR-195 target gene

**Figure 4:**
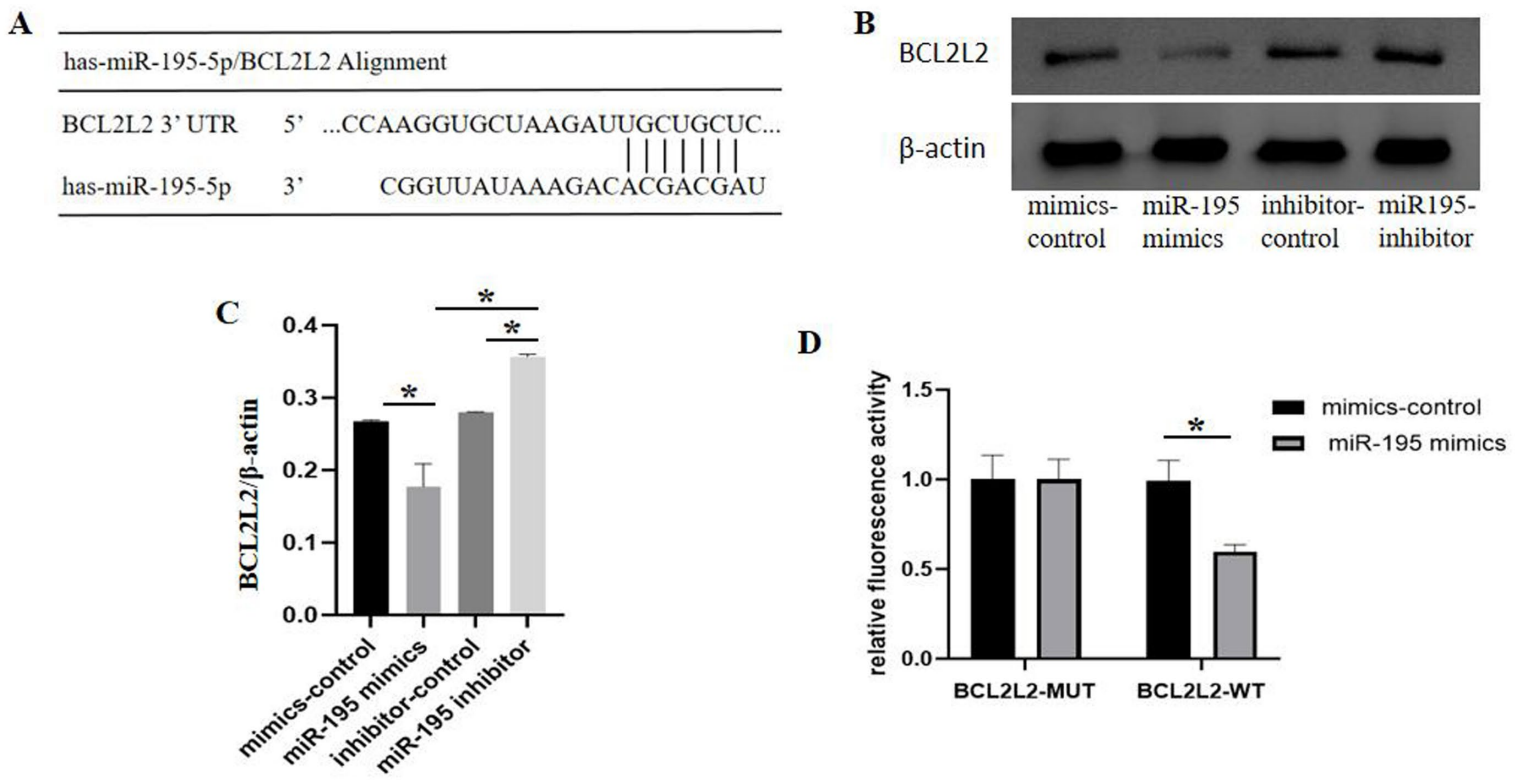
MIR-195 negatively regulated BCL2L2 expression. **(A)** Predicted binding site of miR-195 and BCL2L2. **(B)** Protein expression of BCL2L2 in bEnd.3 cells measured by western blot analysis. **(C)** Quantitative analysis of BCL2L2 protein levels (*n* = 3, mean ± SEM). **(D)** Luciferase activity of BCL2L2-WT and BCL2L2-MUT determined by dual-luciferase reporter assay (*n* = 5, mean ± SEM). *p < 0.05 between the two indicated groups. *n*, number of cell samples.

To uncover the underlying mechanism through which miR-195 protects bEnd.3 cells from OGD/R-induced injury, we used a target searching parameter^2^ to identify the targets of miR-195. Among the predicted targets, BCL2L2, which is an important regulator of apoptosis, attracted our interest. The 3′-UTR of the BCL2L2 mRNA possesses a binding site for miR-195 (Figure 4A). Moreover, to examine the regulatory effect of miR-195 on BCL2L2 expression, bEnd.3 cells were transfected with miR-195 mimics. As expected, miR-195 overexpression significantly decreased BCL2L2 protein levels in bEnd.3 cells (Figures 4B,C). To further investigate whether miR-195 binds to the 3′-UTR region of the BCL2L2 mRNA, a psiCHECK vector containing the 3′-UTR fragment of BCL2L2 was constructed, and a luciferase assay was performed in HEK293 cells. As expected, miR-195 mimics significantly suppressed luciferase activity in the BCL2L2-WT group, whereas it had no effect on luciferase activity in the BCL2L2-MUT group (Figure 4D). These results confirm that BCL2L2 is a target gene of miR-195.

### miR-195 regulates cellular barrier function by targeting BCL2L2

**Figure 5:**
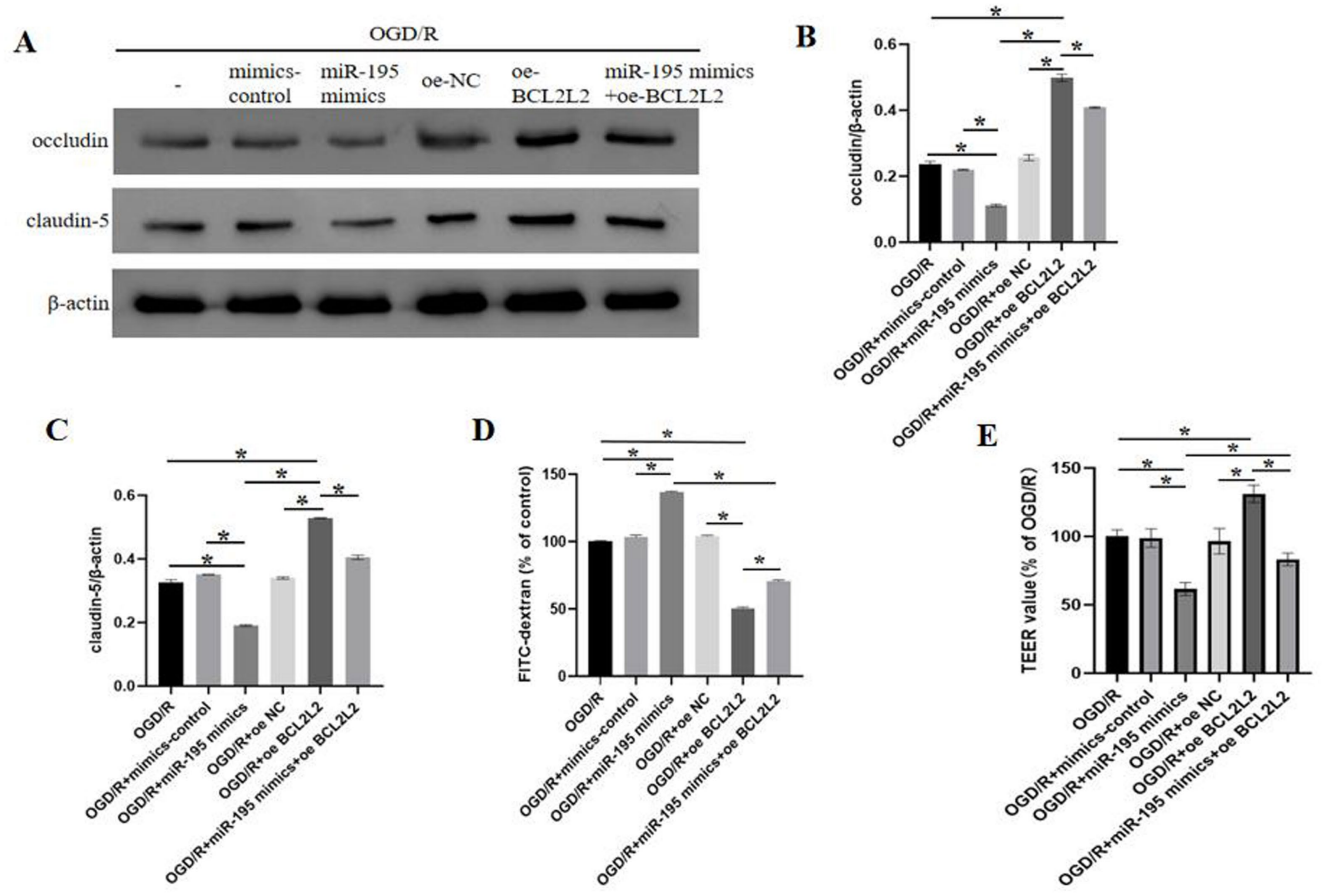
Mi-195 exacerbated OGD/R-induced BBB disruption and TJP degradation by targeting BCL2L2. **(A)** Western blot analysis showed that miR-195 treatment decreased the expression of occludin and claudin-5. **(B)** Quantitative analysis of occludin protein levels (*n* = 3, mean ± SEM). **(C)** Quantitative analysis of claudin-5 protein levels (*n* = 3, mean ± SEM). **(D)** Permeability of bEnd.3 cells was examined using fluorescein isothiocyanate-dextran (*n* = 5, mean ± SEM). **(E)** bEnd.3 cell monolayer permeability was measured using TEER assays (*n* = 5, mean ± SEM). *p < 0.05 compared with the indicated group.

TJPs, particularly occludin and claudin-5, are significant regulators of BBB assembly and function in BMECs (Zhao et al., 2022; Langen et al., 2019). To further investigate the mechanism of miR-195 in the BBB under *in vitro* conditions, bEnd.3 cells were subjected to OGD/R treatment. The expression levels of occludin and claudin-5 were assessed using western blot analysis and immunofluorescence staining. It has been established that occludin and claudin-5 expressions are markedly reduced in bEnd.3 cells following OGD/R (Zhang et al., 2023; Li and Tian, 2021). Compared to the OGD/R group, overexpression of BCL2L2 (oe-BCL2L2) attenuated the degradation of occludin and claudin-5, whereas miR-195 mimics promoted their degradation. Notably, co-transfection with miR-195 mimics and oe-BCL2L2 partially reversed the effects induced by miR-195 mimics alone (Figures 5A–C). Further supporting these findings, TEER and FITC-dextran (40 kDa) permeability assays showed that miR-195 increased the degradation of occludin and claudin-5, an effect that could be partially rescued by oe-BCL2L2 (Figures 5D,E).

### miR-195 regulates cell apoptosis by targeting BCL2L2

**Figure 6:**
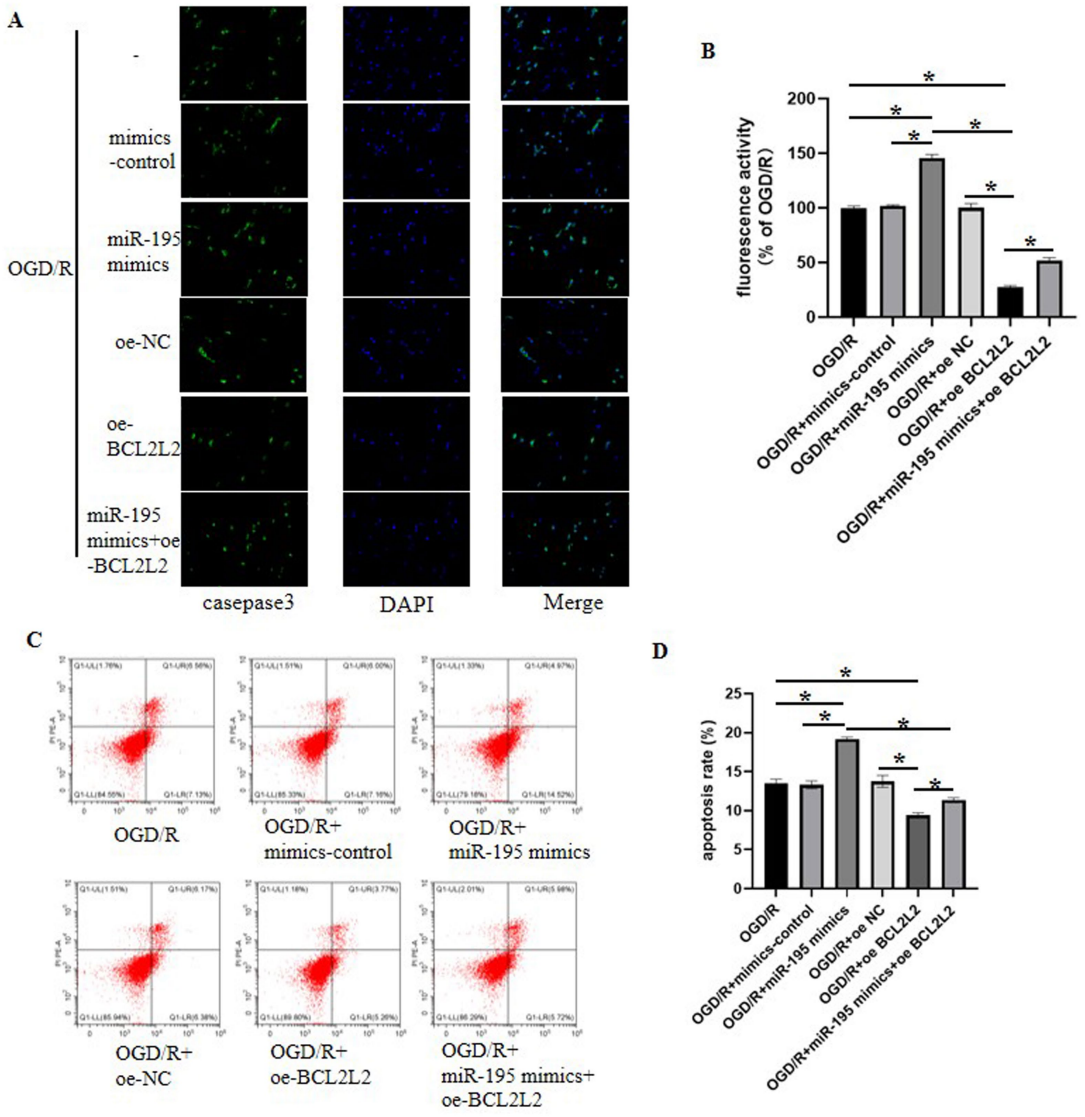
MIR-195 can promote the apoptotic rate of OGD/R-treated bEnd.3 cells by downregulating BCL2L2 expression. **(A)** Expression of caspase-3 analyzed by immunofluorescent staining. **(B)** Quantification of the fluorescence intensity of caspase-3 (*n* = 5, mean ± SEM). **(C)** Annexin V and propidium iodide double-staining of bEnd.3 cells, analyzed by flow cytometry. **(D)** Quantitative analysis of apoptosis in Annexin V/PI-stained cells by flow cytometry (*n* = 5, mean ± SEM).

To further investigate the mechanisms through which miR-195 regulates apoptosis in bEnd.3 cells, cellular apoptosis was assessed by measuring the fluorescence intensity of caspase-3 and Annexin V following OGD/R treatment. Both assays demonstrated that oe-BCL2L2 reduced OGD/R-induced apoptosis, whereas miR-195 mimics promoted it. Compared to the OGD/R group, caspase-3 fluorescence intensity increased in the OGD/R + miR-195 mimic group but decreased in the OGD/R + oe-BCL2L2 group. Moreover, the pro-apoptotic effect of miR-195 mimics was reversed in the group co-transfected with miR-195 mimics and oe-BCL2L2 (Figures 6A,B). The flow cytometry results were consistent with those from the caspase-3 fluorescence intensity assay. Specifically, compared to the OGD/R group, apoptosis was enhanced in the OGD/R + miR-195 mimic group but attenuated in the OGD/R + oe-BCL2L2 group. Similarly, co-transfection with miR-195 mimics and oe-BCL2L2 abolished the pro-apoptotic effect induced by miR-195 mimics (Figures 6C,D).

## Discussion

Ischemic stroke is a major cause of brain injury, and its treatment remains challenging (Ho and Powers, 2025; Paul and Candelario-Jalil, 2021). The disruption of BBB structure and function following acute ischemic stroke contributes to a worsened prognosis through the induction of edema and hemorrhage (Alonso-Alonso et al., 2022; Nian et al., 2020). In the present study, we found that downregulation of miR-195 expression exerted a protective effect against OGD/R-induced endothelial barrier injury. Further investigation revealed that downregulation of miR-195 reduced apoptosis in bEnd.3 cells by enhancing BCL2L2 expression.

BMECs form the BBB through TJPs and maintain its function (Kadry et al., 2020). Previous studies have shown that BBB disruption in ischemic stroke is associated with apoptosis of bEnd.3 cells (Huang et al., 2022; Li et al., 2023). The role of miR-195 in cell apoptosis remains controversial. miR-195-5p suppresses apoptosis induced by intracerebral hemorrhage through the inhibition of MMP-9/MMP-2 expression (Tsai et al., 2024). Overexpression of miR-195-5p alleviates apoptosis caused by cerebral I/R injury via the PTEN-AKT signaling pathway (Ren et al., 2021). Furthermore, in an OGD/R model, miR-195 alleviated apoptosis by suppressing the IKKα-mediated NF-κB pathway (Yang et al., 2021). However, substantial evidence also indicates that miR-195 can promote apoptosis in different models. For instance, miR-195 enhances hypoxia/reoxygenation-induced cardiomyocyte apoptosis by downregulating c-myb (Chen et al., 2016). Similarly, miR-195-5p inhibits neuronal cell proliferation and accelerates apoptosis by targeting GFRA4 (Wang et al., 2021). Inhibition of miR-195 reduces hypoxia-induced cardiomyocyte apoptosis by targeting PPARγ and FASN (Lin et al., 2021). In our study, miR-195 exerted a pro-apoptotic effect on bEnd.3 cells under OGD/R conditions, as confirmed by flow cytometry and TUNEL staining. These observations suggest that miR-195 may function either as a pro-apoptotic or anti-apoptotic factor. The role of miR-195 in apoptosis is highly complex and likely depends on cell type, specific context, and the apoptotic signaling pathways involved.

BCL2L2, a member of the Bcl2 family, is located at band q11.2-q12 on human chromosome 14 and serves as a critical regulator of cell death. It encodes the BCL-W protein, whose promoter region is highly conserved among humans, mice, and rats (Uittenbogaard et al., 2009; Gibson et al., 1996). Previous studies have suggested that BCL-W may play a significant role in regulating neuronal death following ischemic brain injury (Yin et al., 2010; Sun et al., 2003). Its anti-apoptotic mechanism is associated with the inhibition of the death effectors BAX and BAK (Mansour et al., 2025; Czabotar et al., 2014).

The mechanism through which miR-195 regulates apoptosis in bEnd.3 cells may be mediated through the suppression of downstream anti-apoptotic proteins. Target gene prediction using TargetScan (see text footnote 1) identified complementary binding sites between miR-195 and BCL2L2. Western blot analysis showed that inhibition of miR-195 promoted BCL2L2 expression, while overexpression of miR-195 downregulated BCL2L2 expression. Subsequent dual-luciferase reporter assay revealed a direct targeting interaction between miR-195 and BCL2L2. Both western blot and dual-luciferase reporter results suggested that BCL2L2 is a target gene of miR-195. MiR-195 may affect the homeostasis of the BBB after I/R injury by reducing the expression of BCL2L2.

TJPs, such as occludin and claudin-5, are known to play crucial roles in regulating BBB permeability during ischemic stroke (Zheng et al., 2023; Abdullahi et al., 2018). Previous studies have indicated that apoptosis can induce the endocytosis and degradation of TJPs (Bojarski et al., 2004). Attenuation of apoptosis may contribute to the preservation of BBB integrity by preventing the degradation of TJPs. In our study, we found that miR-195 promoted apoptosis in bEnd.3 cells, increased cellular barrier permeability, and further enhanced the degradation of occludin and claudin-5. These effects were reversed by overexpression of BCL2L2. Therefore, we speculate that the underlying mechanism through which BCL2L2 partially reverses miR-195-mediated exacerbation of OGD/R-induced apoptosis and BBB disruption involves its anti-apoptotic function.

Here, we provide the first evidence that downregulation of miR-195 exerts a protective effect against BBB disruption following OGD/R, suggesting that the miR-195/BCL2L2 pathway might be a potential therapeutic target for ischemic stroke.

## Data Availability

The original contributions presented in the study are included in the article/supplementary material, further inquiries can be directed to the corresponding author.

## Footnotes

1
http://www.targetscan.org/vert_72/

2
www.targetscan.org
